# Clusters of Comorbidities in Multiple Sclerosis and Their Influence on Healthcare Resource Usage

**DOI:** 10.1111/ene.70386

**Published:** 2025-11-05

**Authors:** Simón Cárdenas‐Robledo, Susana Otero‐Romero, Juan David García, Nuria López, Pere Carbonell‐Mirabent, Marta Rodríguez, Claudia Guío‐Sánchez, René Carvajal, Maria Angels Passarell‐Bacradit, Jaume Sastre‐Garriga, Xavier Montalban, Mar Tintoré

**Affiliations:** ^1^ Centro de Esclerosis Múltiple (CEMHUN), Hospital Universitario Nacional de Colombia, Departamento de Medicina Interna, Facultad de Medicina Universidad Nacional de Colombia Bogotá Colombia; ^2^ Faculty of Medicine University of Vic‐Central University of Catalonia (UVic‐UCC) Vic/Manresa Catalonia Spain; ^3^ Neurology‐Neuroimmunology Service, Multiple Sclerosis Center of Catalonia (Cemcat) Hospital Universitari Vall D'hebron Barcelona Spain; ^4^ Preventive Medicine and Epidemiology Service Hospital Universitari Vall D'hebron Barcelona Spain; ^5^ Departamento de Imágenes Diagnósticas, Facultad de Medicina Universidad Nacional de Colombia Bogotá Colombia; ^6^ Cognitive Impairment and Dementia Unit, Hospital Atenció Intermedia Mutuam Güell Grup Mutuam Barcelona Spain; ^7^ Atención Primaria/IDIAP Jordi Gol Primary Care Research Institute Institut Catalá de la Salut Barcelona Spain

**Keywords:** cardiovascular diseases, clustering, comorbidity, healthcare resource usage, latent class analysis, mental disorders, multiple sclerosis

## Abstract

**Introduction:**

MS patients are at increased risk of comorbidities and use more healthcare resources. Multimorbidity approached as the number of conditions is flawed by classifying patients with different needs as equal. We aimed to explore how comorbidities cluster and their impact on healthcare resource usage.

**Methods:**

We used latent‐class models of up to 10 clusters in a population‐based sample of MS patients. The optimal number of clusters was determined using model metrics and similarity/entropy measures, and cluster stability was assessed by bootstrapping. Sociodemographic characteristics and healthcare‐resource usage according to the clusters assigned were compared to each other and to patients without comorbidities using univariable and adjusted linear regression models.

**Results:**

In 5548 MS cases, of which 60% had comorbidities, the optimal number of comorbidity clusters was two, comprising a high frequency of cardiovascular comorbidities and psychiatric disorders. Patients in the cardiovascular cluster were older, and in the psychiatric cluster were more frequently female. After adjusting for sociodemographic variables, healthcare resource usage was higher for patients with comorbidities, particularly for nurse (1.1 more average yearly visit; [95% CI 0.41–1.8]; *p* = 0.002), primary care (1.8 more visits; [95% CI 1.4–2.1]; *p* < 0.001), and medication dispensation (336 more dosage units; [95% CI 260–402]; *p* < 0.001) in the cardiovascular cluster, and annual sick‐leave days (3.8 more days; [95% CI 0.25–7.3]; *p* = 0.036) in the psychiatric cluster.

**Discussion:**

We observed clustering of comorbidities around cardiovascular comorbidities and mental disorders, which impacted healthcare resource usage differently. Further research is needed to assess the influence of these clusters on the prognosis of MS.

## Graphical abstract

Using latent‐class models in a population‐based sample of MS patients, cardiovascular and psychiatric clusters were identified. Nurse visits and medication dispensation were higher in the cardiovascular cluster, and annual sick‐leave days were higher in the psychiatric cluster.

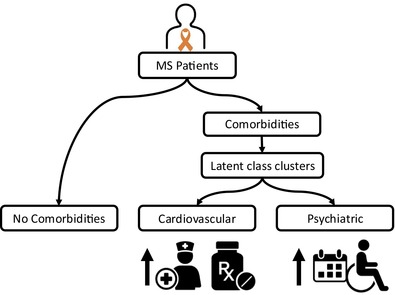

## 1 Introduction

Multiple Sclerosis (MS), the leading cause of neurological disability in young adults in developed countries, is a chronic, inflammatory and degenerative disease of the central nervous system [1]. Comorbidities are frequent in MS patients [2] and have been associated with a worse prognosis [3, 4, 5] and mortality [6, 7]. MS itself imposes an important economic burden on healthcare systems [8, 9], over which the healthcare consumption related to comorbidities is added [10, 11, 12]. Therefore, from the societal standpoint, the identification of the comorbidities most likely to increase the usage of these finite resources is important for their appropriate management. The issue of co/multimorbidity has been traditionally addressed by describing the number of comorbid diseases, or with operational definitions based on the number of conditions [13, 14]. Although adequate for large population studies and certain outcomes such as mortality [15], this approach is potentially flawed due to the possibility of classifying patients with different prognoses and needs as equal (e.g., a patient with only cancer and another with only migraine are considered in the same group of one comorbidity) and thus does not reflect on the specific needs of the patients or on the interactions between comorbidities [16, 17]. Population‐based studies on multimorbidity in primary care have used several approaches to classify patients not only in terms of the number of comorbidities, but on their pattern of presentations [18]. These studies have used different clustering techniques, and examined the influence of comorbidity clusters on outcomes such as mortality [19, 20] and healthcare resource usage [19]. This approach, however, has been scarcely studied in MS patients. A recent study [21] applied a latent‐class clustering method in a sample of the Australian MS longitudinal study and found that comorbidity distribution was better described with five clusters, which were named the minimally diseased class, the metabolic class, the mental health–allergy class, the nonmetabolic class, and the severely diseased class. The clusters differed in age, MS duration and disability. The distribution of comorbidities differs across the different populations and according to the information sources (clinic‐based vs. population‐based) [22, 23], and so does potentially any clustering. Moreover, different clustering of comorbidities might impact the use of healthcare resources. Clusters of multimorbidity have been defined using several techniques [24, 25] and validated by different means, including model and distance metrics [19, 26], observed/expected frequency of conditions [27, 28, 29], clinical judgement [19, 30], and outcomes themselves [19, 31], among others. Therefore, we aimed to describe how comorbidities from a population‐based sample of MS patients cluster, and to validate these clusters by assessing their influence in healthcare resource usage.

## 2 Materials and Methods

This is an observational cross‐sectional population‐based case control study of patients within the primary care network of Catalonia, in northeastern Spain [11]. At the time of the database lockup in 2016, Catalonia had a population of nearly 7.5 million, 20% of which lived in the capital, Barcelona. Details of the data sources and quality controls have been described previously [11]. The primary care research system gathers anonymized longitudinal data from patients cared for by the Catalan Institute of Health since 2006, from the basic care units with higher standards of data quality [32]. These are units treating more than 500 subjects, which are in the highest quintile of a quality score that compares the observed and expected frequencies of common health problems [32]. The database covers a representative 80% of the general population in Catalonia [33]. Subjects included were patients over 18 years of age, with an ICD‐10 diagnostic code for MS (G35), who had at least one visit in the primary care network between January 2006 and October 2016 [11]. This study was approved by the ethics committee of the Foundation University Institute for Primary Health Care Research Jordi Gol, with a waiver for informed consent.

### 2.1 Variables

#### 2.1.1 Demographic and Clinical Variables

Basic demographic (sex, age, province of residence, and socioeconomic status [SES]) and clinical variables (MS duration—based on the date of the ICD codification—and smoking) were extracted.

#### 2.1.2 Comorbidities

The presence of selected comorbidities registered by primary health physicians using ICD‐10 codes. The selection of comorbidities was based on their frequency reported in the literature for MS patients [2] and their overall frequency in the general population and was done using the highest code in the hierarchical system of the ICD‐10 (see [11] for details and Supporting Information).

#### 2.1.3 Outcomes

The main outcome variables were the mean annual nurse and primary care physician (PCP) visits, mean annual sick leave days (only for patients not on disability pension), and the annual overall medication dispensations. The latter were measured as the total number of dosage units (in this case, tablets) of medications delivered to each patient annually, regardless of the indication. For example, if a patient with hypertension was prescribed a beta‐blocker twice daily but only got the medications corresponding to 8 months, then the number of dispensations corresponds to 480 (60 tablets/month for 8 months).

### 2.2 Statistical Analysis

#### 2.2.1 Comorbidity Clustering

The clustering of comorbidities was analysed using latent class (LC) models. This is a probabilistic approach that assigns to each observation in a dataset a membership to an unobserved (latent) class, based on the variation of manifest indicators (in this case, the presence or absence of comorbidities) [34]. This technique was chosen over other clustering methods (particularly over distance‐based methods) for several reasons: comorbidities are binary and asymmetric variables (i.e., two subjects sharing one comorbidity are likely to be more similar than two patients who do not have that condition); LC models are exhaustive and exclusive (i.e., each and every observation is assigned in only one latent class), and subject to model diagnostics with usual metrics; LC models have shown better performance than other clustering methods in terms of stability and reproducibility [35]. For the cluster analysis, only subjects with comorbidities were selected. Patients without comorbidities were considered a cluster in themselves and were later incorporated for the outcome analysis (Figure S1a).

LC models were built without a priori considerations regarding the number of classes, although they were limited to 10 for the sake of clinical usefulness, parsimony, and interpretability. For the construction of the models, we included the information gathered on all the 26 comorbidities (Figure S1b). For each model, we calculated several metrics of model and cluster behaviours [36] that were then used for selecting the optimal number of clusters. First, the Bayesian information criterion (BIC), which compares how well the models fit to the data. Second, we used the integrated completed likelihood (ICL), a measure that combines the BIC with the entropy of the models. The ICL is particularly useful for identifying clusters that are most distinct from one another. When comparing models, those with the lowest values of BIC and ICL were deemed to have the best behaviour. Finally, the average silhouette width (ASW), which is a dissimilarity measure used for selecting the number of clusters when using distance‐based methods. The ASW assesses how well the clusters are defined by measuring the compactness of the clusters (how similar are the observations within a cluster to each other) and the separation between clusters (how dissimilar are the values from one cluster to those of other clusters). The ASW has possible values from −1 to 1, higher values indicating a better definition of the clusters (Figure S1c). These three metrics were estimated for each model, and the best model according to each was then assessed for stability by bootstrapping (Figure S1d). This was done by selecting 100 random subsamples from the dataset and estimating the Jaccard coefficient (JC) and the adjusted Rand index for each of the partitions derived from the models in each pair of subsamples. The JC calculates the relation between the common number of observations assigned to one cluster in two subsamples and the total number of observations of the two subsamples. The adjusted Rand index (ARI) measures how similar a partition of a dataset is to a partition of another dataset (here of two of the subsamples). Both parameters measure the similarity of two sample sets and range from 0 (no similarity at all) to 1 (complete similarity) [37]. Then, for each model, the values of ARI and JC of each subsample were averaged and the model with the highest average values (i.e., highest stability) was selected for the outcome analysis (Figure S1e). Based on the latter, the discriminative power of each comorbidity for the clustering in the final model was then estimated. This is a measure of the relation of the probability that each variable is relevant or irrelevant for the clustering [38]; the higher the value obtained for each variable, the more relevant it is for the cluster definition. Finally, the patients with no comorbidities were included as a separate cluster for the outcome assessment (shown in Figure S1f).

#### 2.2.2 Outcome Assessment and Variable Distribution According to Clusters

Demographic and clinical variables were described according to the selected clusters, using the group without comorbidities as the reference for comparisons. Quantitative variables were described using means and standard deviations (SD) or medians and interquartile ranges (IQR) according to their statistical distributions, and qualitative variables were described in terms of absolute and relative frequencies. Comparisons were done with the student's t‐test, the Kruskal‐Wallis rank sum test, or Pearson's Chi‐squared test as appropriate.

The influence of the selected comorbidity clusters on the outcome variables was assessed by direct comparisons using Cohen's d, as well as uni‐ and multivariable linear regression models, which included sex, age, and SES as covariates.

Cohen's d was used as a measure of effect size to perform comparisons in the outcome variable to avoid the effect of the large sample size in *p*‐values. The effect size using Cohen's d is commonly interpreted as small (0.2), medium (0.5), and large (0.8) [39].

Statistical analysis was done using R version 4.0.2 [36, 38, 40].

## 3 Results

### 3.1 Demographic and Clinical Variables

A total of 5548 MS patients were analysed, of which 3863 (69.6%) were women. Mean (SD) age and MS duration were 48.3 (12.8) and 12.0 (7.6) years, respectively. The mean (SD) number of comorbidities was 1.1 (1.22). At least one comorbidity was found in 3334 (60.1%) of the subjects; 1685 (30.4%) had at least two, and 718 (12.9%) had at least three comorbidities.

### 3.2 Cluster Selection and Description

**FIGURE 1:**
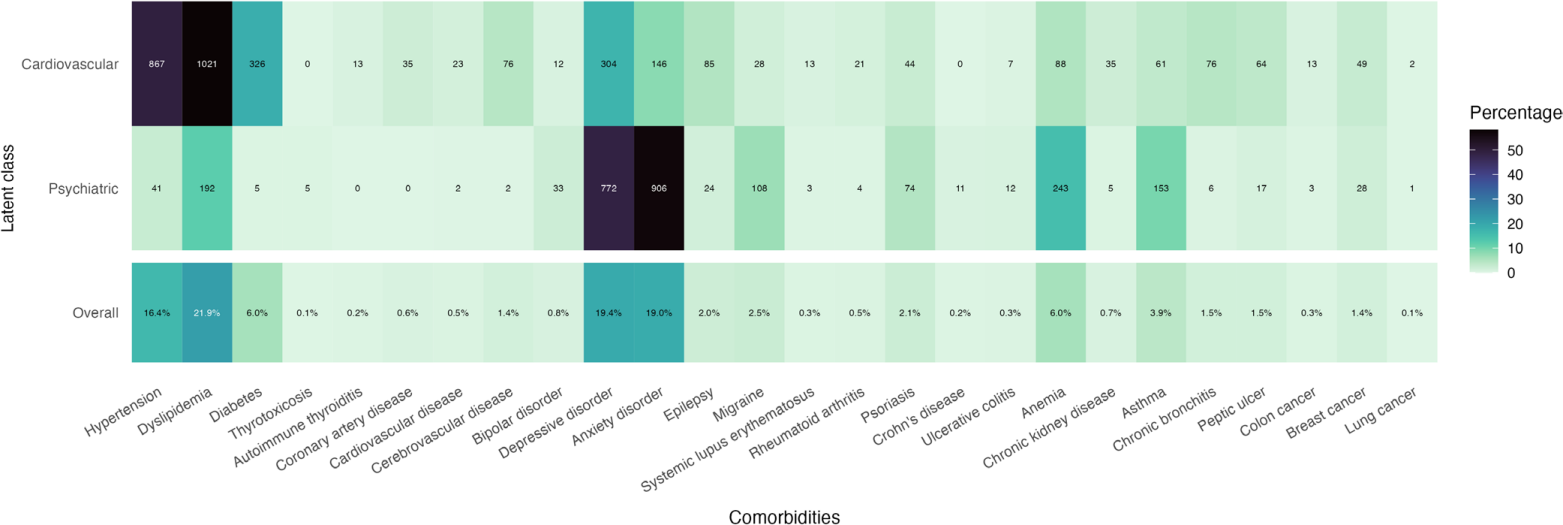
Heatmap showing the frequency of individual comorbidities in patients assigned to the two clusters identified (top rows) and in the whole sample (bottom row). Brighter colours indicate lower and darker colours indicate higher frequencies of the selected comorbidities within each cluster. Clusters were labelled according to the frequency of the most common comorbidities within each cluster.

The process of LC model selection yielded three options for the analysis of cluster stability (Table S1). The lowest BIC and ICL values were found for the model with two clusters, and the highest ASW was for the model with eight clusters. We then assessed the stability of these two models, which yielded JC of 0.85 and 0.70, and ARI of 0.83 and 0.78, respectively. Thus, the final LC model selected was the one with two clusters. The discriminative power of the different comorbidities for the model with two clusters was highest for hypertension and anxiety disorders and lowest for lung cancer (Figure S2). Based on the discriminative power of each variable for the model with two clusters and on the frequency of the individual comorbidities among each cluster (Figure 1), the two clusters were labelled and will be henceforth referred to as “Cardiovascular” and “Psychiatric”.

### 3.3 Demographic Variables and Outcomes According to Selected Clusters

The demographic and clinical variables according to the clusters are described in Table 1. The clusters differed from each other and from patients without comorbidities. Patients within the cardiovascular cluster were older (mean age 56.3 years; SD: 11.9) than those in the psychiatric cluster (mean age 45.6 years; SD: 11.0) (*p* < 0.001) and without comorbidities (mean age 44 years; SD: 11.7) (*p* < 0.001). Women were more frequently encountered in the psychiatric cluster (80%) when compared to the cardiovascular cluster (65%) (*p* < 0.001) and those without comorbidities (66%) (*p* < 0.001). MS duration was longer in the cardiovascular cluster when compared to patients without comorbidities (*p* < 0.001). No significant differences were found between the two clusters in terms of SES (*p* = 0.947), but a higher frequency of ever smokers (57%) was found in the psychiatric cluster compared to patients without comorbidities (49%) (*p* < 0.001) and the cardiovascular cluster (47%) (*p* < 0.001).

**TABLE 1:** Demographic and clinical characteristics of the identified clusters.

Characteristic		No comorbidities *n* = 2214	Cardiovascular *n* = 1754	Psychiatric *n* = 1580	*p* (cardiovascular vs. no comorbidities)	*p* (psychiatric vs. no comorbidities)
Age years, *n* (%)	< 40	860 (38.8%)	149 (8.5%)	490 (31.0%)	< 0.001^a^	< 0.001^a^
	40–59	1111 (50.2%)	889 (50.7%)	918 (58.1%)		
	60–79	233 (10.5%)	667 (38.0%)	162 (10.3%)		
	> 80	10 (0.5%)	49 (2.8%)	10 (0.6%)		
Sex female, *n* (%)		1470 (66.4%)	1136 (64.8%)	1257 (79.6%)	0.298^a^	< 0.001^a^
Disease duration years, mean (SD)		11.2 (7.1)	13.4 (8.6)	11.6 (6.9)	< 0.001^b^	0.130^b^
Socioeconomic status *n* (%)^c^	Q1	282 (17.0%)	197 (14.8%)	185 (14.7%)	0.061^a^	0.014^a^
	Q2	415 (25.0%)	316 (23.8%)	293 (23.3%)		
	Q3	424 (25.5%)	324 (24.4%)	297 (23.6%)		
	Q4	328 (19.7%)	278 (20.9%)	279 (22.2%)		
	Q5	212 (12.8%)	213 (16.0%)	205 (16.3%)		
Ever smoker *n* (%)^d^		859 (48.7%)	783 (47.0%)	804 (56.6%)	0.321^a^	< 0.001^a^

^a^ Pearson's Chi‐squared test.

^b^ Student's *t*‐test.

^c^ Data available for 4284 (76.6%) subjects (No comorbidities: 1661, Psychiatric cluster:1259, Cardiovascular cluster: 1328).

^d^ Data available for 4840 (87.4%) subjects (No comorbidities: 1863, Psychiatric cluster: 1420, Cardiovascular cluster: 1667).

**FIGURE 2:**
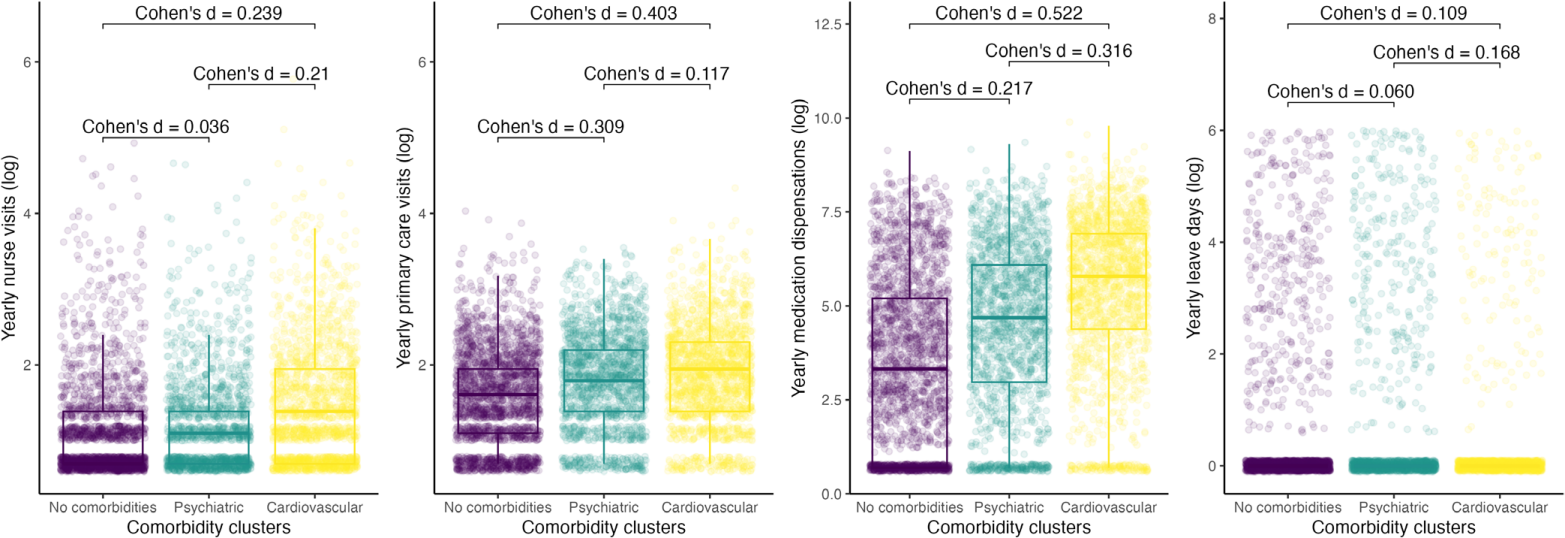
Boxplots depicting healthcare resource usage among patients without comorbidities and the cardiovascular and psychiatric clusters. Cohen's d for the estimation of the effect size of each comparison is shown.

Patients in the cardiovascular cluster had more frequent nurse visits (mean annual visits 4.5; SD 11.8) and PCP visits (mean annual visits 6.1; SD 5.9) than patients in the psychiatric cluster (mean nurse visits 2.5; SD 6.7 and mean PCP visits 5.4; SD 5.9) and patients without comorbidities (mean nurse visits 2.2; SD 7.6 and mean PCP visits 3.9; SD 4.7) (*p* < 0.001). Medication dispensations were also higher in the cardiovascular cluster (mean 749.1; SD 1177) compared to the psychiatric cluster (mean 426.1; SD 815.7) and patients without comorbidities (mean 270.6; SD 637.6) (*p* < 0.001). Annual sick leave days were higher for the psychiatric cluster (mean 14.7; SD 54.6) compared to the cardiovascular cluster (mean 6.9; SD 36.8) and those without comorbidities (mean 11.6; SD 47.6) (*p* < 0.001). The effect size of the comorbidity clusters was moderate for the comparison between the cardiovascular cluster and patients with no comorbidities in terms of PCP visits (Cohen's d = 0.403) and medication dispensations (Cohen's d = 0.522). For the remainder of the comparisons, the effect size was small (Figure 2).

The differences remained significant after adjusting for sex, age, and SES in regression models only for some of the comparisons (Table 2). In terms of clinical visits, patients in the cardiovascular cluster had, on average, one more nurse visit (*β* = 1.1; 95% CI = 0.41–1.8; *p* = 0.002) and nearly two more PCP visits (*β* = 1.8; 95% CI = 1.4–2.1; *p* < 0.001) per year, as compared to patients without comorbidities. Patients in the psychiatric cluster had also more PCP annual visits (*β* = 1.4; 95% CI = 1.0–1.7; *p* < 0.001) than patients without comorbidities. Patients in the psychiatric cluster had, on average, nearly four more annual sick leave days compared to patients with no comorbidities (*β* = 3.8; 95% CI = 0.25–7.3; *p* = 0.036), and both psychiatric (*β* = 126; 95% CI = 60–192; *p* < 0.001) and cardiovascular (*β* = 331; 95% CI = 260–402; *p* < 0.001) clusters had significantly more medication dispensation than patients without comorbidities.

**TABLE 2:** Univariable and adjusted linear regression models comparing healthcare resource usage.

Characteristic			Annual nurse visits		Annual PCP visits		Annual sick‐leave days		Annual medication dispensations	
			*β* (95% CI)	*p*	*β*	*p*	*β*	*p*	*β*	*p*
Univariable	Comorbidity clusters^a^	Psychiatric	0.26 (−0.32 to 0.84)	0.4	1.5 (1.1 to 1.8)	< 0.001	3.0 (0.04 to 6.1)	0.047	155 (98 to 213)	< 0.001
		Cardiovascular	2.3 (1.8 to 2.9)	< 0.001	2.1 (1.8 to 2.5)	< 0.001	−4.7 (−7.6 to −1.8)	0.002	478 (423 to 534)	< 0.001
Multivariable	Comorbidity clusters^a^	Psychiatric	0.06 (−0.60 to 0.72)	0.9	1.4 (1.0 to 1.7)	< 0.001	3.8 (0.25 to 7.3)	0.036	126 (60 to 192)	< 0.001
		Cardiovascular	1.1 (0.41 to 1.8)	0.002	1.8 (1.4 to 2.1)	< 0.001	0.68 (−3.1 to 4.5)	0.7	331 (260 to 402)	< 0.001
	Age^b^		0.09 (0.07 to 0.11)	< 0.001	0.03 (0.02 to 0.05)	< 0.001	−0.40 (−0.53 to −0.28)	< 0.001	16 (13 to 18)	< 0.001
	Sex^c^		−0.50 (−1.1 to 0.08)	0.092	−0.38 (−0.71 to −0.06)	0.021	0.81 (−2.3 to 3.9)	0.6	17 (−42 to 76)	0.6
	Socioeconomic status^d^	Q2	0.93 (0.06 to 1.8)	0.036	1.1 (0.61 to 1.6)	< 0.001	4.0 (−0.64 to 8.6)	0.091	−78 (−165 to 9.3)	0.080
		Q3	0.85 (−0.01 to 1.7)	0.053	0.88 (0.40 to 1.4)	< 0.001	2.8 (−1.8 to 7.4)	0.2	−95 (−182 to −8.4)	0.031
		Q4	1.3 (0.41 to 2.2)	0.004	1.1 (0.56 to 1.6)	< 0.001	3.2 (−1.6 to 8.0)	0.2	−57 (−147 to 33)	0.2
		Q5	1.4 (0.38 to 2.3)	0.006	1.7 (1.1 to 2.2)	< 0.001	7.1 (1.9 to 12)	0.007	8.1 (−89 to 106)	0.9

^a^ No comorbidities as reference.

^b^ Change per each 1 year increase in age.

^c^ Male sex as reference.

^d^ The quintile with the least deprivation as reference.

## 4 Discussion

In this population‐based observational study, we provide evidence that comorbidities in MS patients tend to cluster around cardiovascular conditions (hypertension, dyslipidaemia, and diabetes) and psychiatric diseases (anxiety and depressive disorders), and that these clusters have different demographic characteristics and different behaviours of healthcare‐resource usage.

Our results show that MS patients with cardiovascular comorbidities were older than those without and with psychiatric comorbidities, and that the latter were more frequently female and ever smokers. The healthcare resource usage was higher in patients with comorbidities, and its pattern differed across the comorbidity clusters, even after adjusting for socio‐demographic variables. We found a higher frequency of nurse and PCP visits and higher medication dispensations among cases in the cardiovascular cluster when compared to both the psychiatric cluster and those patients without comorbidities.

Some characteristics of the clusters deserve attention. The psychiatric cluster is composed mainly of women, which is in line with the higher frequency of depression and anxiety in women in the general population [41], but disagrees with previous observations in MS, where no significant differences in the frequency of depression in both sexes have been reported [42, 43, 44]. Patients in the cardiovascular cluster were found to be significantly older, which may reflect the increased frequency of cardiovascular comorbidities in elderly patients with MS [45, 46]. Patients in the psychiatric cluster were more frequently ever smokers than those without comorbidities and those in the cardiovascular cluster. This could be accounted the known association of depression and/or anxiety with smoking in patients with MS [47, 48], in turn explained by an increased overall proclivity of people with these disorders to have addictive behaviors such as smoking [49]. Given the known association of smoking with negative outcomes in MS [50], patients with psychiatric comorbidity should be prioritized in smoking cessation programs. The finding that ever smoking was more frequent in the psychiatric than in the cardiovascular cluster speaks to the fact that the cardiovascular cluster was defined primarily by the presence of hypertension and dyslipidemia, and not by the diseases associated with smoking, such as coronary disease and stroke. These were infrequent in our population, and thus had a low discriminative power for defining the clusters. It is noteworthy that patients in the psychiatric cluster require significantly more annual sick leave days than patients in the cardiovascular cluster and patients without comorbidities. This agrees with several observations of increased levels of disability [51, 52] in relation to depressive symptoms. However, another explanation is that MS patients in the cardiovascular cluster are older, and therefore more likely to be in permanent disability pension or retirement, in which case no sick leave days are reported.

Our findings are in line with studies assessing multimorbidity in primary care at a population level in which clustering around mental health and cardio‐metabolic disorders has been consistently reproduced [53]. Other clusters grouping conditions such as recurrent falls/sensory deficits and Parkinson's disease/cognitive decline have also been identified [53]. However, we did not include these conditions in our analysis due to the high frequency of sensory disturbances, falls [54, 55], and cognitive decline [56] that are considered manifestations of MS and not comorbidities in themselves, as well as the very low prevalence of Parkinson's disease in MS patients [21]. Comparability of our results with those studies is therefore limited.

A previous study on the ageing general population in Spain showed that a “metabolic/stroke” cluster of comorbidities used almost twice the number of medical visits and doubled the frequency of hospital admissions in the previous year compared to the healthy cluster [57]. In line with these results, we found that, compared with those without comorbidities, people in the cardiovascular cluster almost doubled and tripled the number of clinical visits and medication prescriptions, respectively. Although this might not seem relevant for the individual patient, at the population level and from the healthcare system perspective, increased healthcare usage in patients with an already burdensome disease is of the utmost importance. This supports the notion that comorbidities in patients with MS should be actively sought and treated from the onset of MS.

Ours differs from the previous study assessing comorbidity clustering in MS [21] in a fundamental way. Our sample is population‐based, and the diagnoses of comorbidities are assigned by physicians from primary care centres with high‐quality reporting, selected by the accuracy of their clinical records. The assessment of comorbidities in the former [21] was the patient's self‐report, which is subject to recall and non‐response bias. Another important difference between both studies is that we could not study the impact of the clusters on MS outcomes such as relapses, disability, and treatment. An increased risk of higher patient‐reported disability was found in the “mental health‐allergy” and the “severely diseased” clusters [21], which is in line with studies investigating the relationship with comorbidities and disability [58].

We find noteworthy that the clusters we found are centered around those comorbidities strongly associated with MS activity and disability. Cardiovascular diseases have been linked with more frequent relapses [5, 59], disability worsening [3, 5, 60, 61] and disease activity on MRI [5]. Psychiatric disorders, particularly depression and anxiety, are linked with a poor prognosis of MS, especially in terms of disability [5, 44, 51, 52], as well as relapses [3]. This, and our finding that comorbidity clusters are associated with increased and different healthcare needs, may impact clinical practice in MS by prioritizing which comorbidities deserve more attention and potentially more aggressive management.

The strengths of our study stem from the large sample with high data quality [32, 33], and highly representative of the general population [33, 62]. Also, the clustering approach used has adequate properties for the analysis of the type of data used [35, 53].

Nevertheless, there are several limitations that need to be acknowledged. The pre‐selection of the comorbidities included based on those previously reported for MS populations may lead to selection bias. The inclusion of comorbidities such as skin disease (both melanoma and non‐melanoma), cervical uterine cancers, other autoimmune conditions and/or infectious diseases, as well as those derived from hospitalizations not included in our analysis may have influenced the clustering in our sample. However, although relevant for MS in terms of therapy selection and monitoring, their frequency is low, and it is possible that their effect on the clusters might be minimal. It is also important to bear in mind that the clusters found in our data could be only artefactual and not a representation of a real biological phenomenon, which is known as the reification fallacy [63]. These clusters might be the reflection of the high frequency of the comorbidities included in them, resulting from ascertaining bias. However, the fact that the healthcare resource usage differed across the clusters both qualitatively and quantitatively speaks to the validity of our findings. This is in line with studies using healthcare resource usage outcomes for cluster validation [26, 64]. However, the lack of clinically relevant outcomes in terms of disability, relapses, mortality [26] or quality of life [65] precludes a more thorough assessment of the impact of the clusters of comorbidities found in our sample.

In conclusion, our study provides evidence that patients with MS tend to cluster around cardiovascular and psychiatric comorbidities, and that these drive different aspects of healthcare resource consumption. Our findings may aid in easily incorporating the information of comorbidities in studies of the prognosis of MS. Further studies are warranted to establish if this pattern is also observed in MS patients from other populations with different genetic and risk factor backgrounds, as well as their impact on MS outcomes.

## Author Contributions

S.O.‐R., J.S.‐G., and M.A.P.‐B. drafted the original proposal and obtained funding. S.C.‐R., J.D.G, and P.C.‐M. performed the analysis. S.C.‐R., S.O.‐R., N.L., and M.T. interpreted the results. S.C.‐R. drafted the manuscript. All authors revised the manuscript and approved the final version.

## Ethics Statement

This study was conducted in accordance with the declaration of Helsinki and was fully approved by the ethics committee of the Foundation University Institute for Primary Health Care Research Jordi Gol with a waiver for informed consent.

## Conflicts of Interest

S.C.‐R. was a 2019–2020 ECTRIMS clinical fellowship awardee; in the past 3 years he has received travel expenses for scientific meetings from Roche, Biopas, Novartis, Merck, and Genzyme; compensation for consulting services or participation on advisory boards from Amgen, Merck, Biogen‐Idec, Sanofi, and Novartis; lecture fees from AstraZeneca, Novartis, Merck, Sanofi, Janssen, Lumex pharma, Thermo Fisher, and Biogen‐Idec; and research support from Biogen‐Idec and Novartis. S.O.‐R. has received speaking and consulting honoraria from Genzyme, Biogen‐Idec, Novartis, Roche, Excemed, and MSD; as well as research support from Novartis. P.C.‐M.'s annual salary is supported by a grant from Biogen to Fundació privada Cemcat towards statistical analysis. C.G.‐S. received an ECTRIMS clinical fellowship in 2022–2023. In the last 3 years has received consulting fees from Novartis, Biogen‐Idec, Merck, Roche and Sanofi‐Genzyme and travel expenses for scientific meetings from Biogen‐Inc and Merck. R.C. is currently being funded by the Vall d'Hebron Institut de Recerca. In 2023 he was awarded a Research Training Programme from the European Charcot Foundation. From 2021 to 2022 he received an ECTRIMS Clinical and Research Fellowship training. He has also engaged in consulting and/or participated as a speaker in events organised by Roche, Novartis, BIIB‐Colombia, Merck, and Sanofi. J.S.‐G. serves as co‐Editor for Europe on the editorial board of Multiple Sclerosis Journal and as Editor‐in‐Chief in Revista de Neurología, receives research support from Fondo de Investigaciones Sanitarias (19/950), and has served as a consultant/speaker for Biogen, Celgene/Bristol Meyers Squibb, Sanofi, Novartis, and Merck. X.M. has received speaking honoraria and travel expenses for participation in scientific meetings, has been a steering committee member of clinical trials, or participated in advisory boards of clinical trials in the past years with Abbvie, Actelion, Alexion, Biogen, Bristol‐Myers Squibb/Celgene, EMD Serono, Genzyme, Hoffmann‐La Roche, Immunic, Janssen Pharmaceuticals, Medday, Merck, Mylan, Nervgen, Novartis, Sandoz, Sanofi‐Genzyme, Teva Pharmaceutical, TG Therapeutics, Excemed, MSIF, and NMSS. M.T. has received compensation for consulting services, speaking honoraria, and research support from Almirall, Bayer Schering Pharma, Biogen‐Idec, Genzyme, Immunic Therapeutics, Janssen, Merck‐Serono, Novartis, Roche, Sanofi‐Aventis, Viela Bio and Teva Pharmaceuticals. Data Safety Monitoring Board for Parexel and UCB Biopharma, Relapse Adjudication Committee for IMCYSE SA. J.D.G.; N.L.; M.R. and M.A.P.‐B. have nothing to disclose.

## Supporting information


**Data S1:** ene70386‐sup‐0001‐Samplecode.R.

**Data S2:** ene70386‐sup‐0002‐supplementarymaterial.docx.

## Data Availability

Anonymized data are available from the corresponding author upon reasonable request. The code for the clustering algorithm and selection is provided in the Supporting Information.
