# Supplementary material for: Clusters of Comorbidities in Multiple Sclerosis and Their Influence on Healthcare Resource Usage
Source: Eur J Neurol. 2025 Nov 5;32(11):e70386. doi: 10.1111/ene.70386 (PMC12587307; doi:10.1111/ene.70386)
Supplement: Supplementary file 2 — Data S2: ene70386‐sup‐0002‐supplementarymaterial.docx. [file ENE-32-e70386-s002.docx]

**Supplementary methods:**

The comorbidities (ICD-10 codes) included for the analysis were: disorders of lipoprotein metabolism and other lipidaemias (E78), diabetes (E14), autoimmune thyroiditis (E06.3), thyrotoxicosis with diffuse goitre (E05.0), essential (primary) hypertension (I10), chronic ischemic heart disease (I25.9), cardiovascular disease (I51.6), other specified cerebrovascular diseases (I67.8), Crohn’s disease (K50.9), ulcerative colitis (K51.9), peptic ulcer (K27.9), bipolar affective disorder (F31.9), major depressive disorder (F32.9), other anxiety disorders (F41), epilepsy (G40.9), migraine (G43.9), systemic lupus erythematosus (L93), rheumatoid arthritis (M06.9), psoriasis (L40.9), unspecified anaemia (D64.9), renal insufficiency (N19), unspecified asthma (J45.9), unspecified chronic bronchitis (J42), malignant neoplasm of colon (C18), malignant neoplasm of breast (C50.9), malignant neoplasm of the bronchus or lung (C34.9).


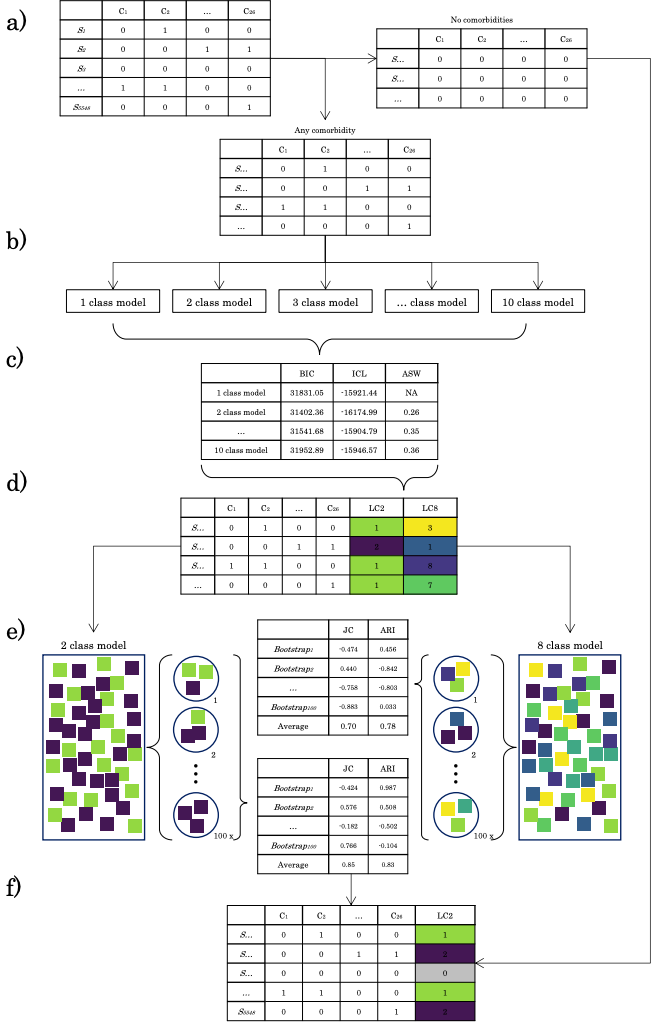
**Supplementary figure 1**: Graphical depiction of the clustering procedure. Subjects with at least one comorbidities were selected for clustering (a), and 10 LC were built in this subsample (b). From each of these models the metrics of fit and cluster behaviour were calculated and compared (c), and the models with the best metrics were selected (d). Each clustering was then assessed by bootstrapping by calculating the stability measures in 100 random subsamples (e), from which the clustering with the higher measures were selected. Finally, the subjects without comorbidities were joined as the no comorbidity cluster (f).

**Supplementary figure 2**: Discriminative power of each comorbidity for the model with two clusters. The influence of each comorbidity in defining each cluster was calculated as the logarithm of the ratio of its probability being relevant or irrelevant for the partition ^21^. Higher estimates indicate higher discriminative power.


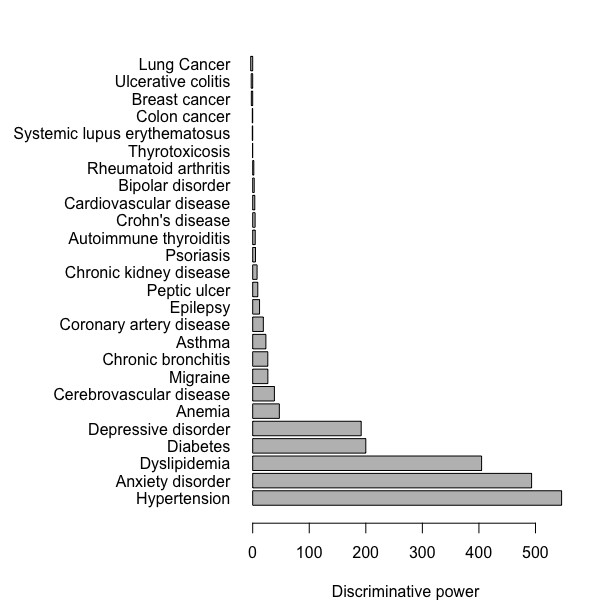


**Supplementary table 1.** Model and cluster metrics

| Number of clusters in the model | Minimum BIC | Minimum ICL | Maximum ASW |
| --- | --- | --- | --- |
| 1 | 31831.05 | -15921.44 | NA |
| 2 | 31402.36* | -16174.99* | 0.26 |
| 3 | 31450.52 | -16093.23 | 0.10 |
| 4 | 31476.56 | -15973.20 | 0.29 |
| 5 | 31449.91 | -15794.32 | 0.31 |
| 6 | 31541.68 | -15904.79 | 0.35 |
| 7 | 31609.60 | -15913.56 | 0.35 |
| 8 | 31710.81 | -15886.37 | 0.36* |
| 9 | 31839.01 | -15947.85 | 0.36 |
| 10 | 31952.89 | -15946.57 | 0.36 |

*The models with the lowest BIC and ICL and the highest ASW were used for the stability analysis
